# Evolving Horizons: Adenovirus Vectors’ Timeless Influence on Cancer, Gene Therapy and Vaccines

**DOI:** 10.3390/v15122378

**Published:** 2023-12-03

**Authors:** Prasad D. Trivedi, Barry J. Byrne, Manuela Corti

**Affiliations:** Department of Pediatrics, University of Florida, Gainesville, FL 32610, USA; prasadtrivedi@ufl.edu (P.D.T.); barry.byrne@ufl.edu (B.J.B.)

**Keywords:** adenovirus, advanced gene therapy, oncolytic virotherapy, vaccine development, regenerative medicine, stem cells, genetic modification, novel viral vectors, clinical trials

## Abstract

Efficient and targeted delivery of a DNA payload is vital for developing safe gene therapy. Owing to the recent success of commercial oncolytic vector and multiple COVID-19 vaccines, adenovirus vectors are back in the spotlight. Adenovirus vectors can be used in gene therapy by altering the wild-type virus and making it replication-defective; specific viral genes can be removed and replaced with a segment that holds a therapeutic gene, and this vector can be used as delivery vehicle for tissue specific gene delivery. Modified conditionally replicative–oncolytic adenoviruses target tumors exclusively and have been studied in clinical trials extensively. This comprehensive review seeks to offer a summary of adenovirus vectors, exploring their characteristics, genetic enhancements, and diverse applications in clinical and preclinical settings. A significant emphasis is placed on their crucial role in advancing cancer therapy and the latest breakthroughs in vaccine clinical trials for various diseases. Additionally, we tackle current challenges and future avenues for optimizing adenovirus vectors, promising to open new frontiers in the fields of cell and gene therapies.

## 1. Introduction

Gene therapy is acclaimed as the 21st century’s medicine. At present, 32 cell and gene therapies have been approved within the United States, including one oncolytic virus [1]. Gene therapy encompasses a range of techniques aimed at modifying gene expression. These methods include replacing a faulty gene with a healthy one, deactivating a malfunctioning gene, and introducing a new or modified gene into the body to address specific diseases. Despite previous challenges, it is a rapidly advancing field exploring innovative strategies for complex health problems. The urgency of COVID-19 vaccines marked a pivotal moment, propelling global advancements in gene therapy, including mRNA and adenovirus (AdV) vectors. By April 2022, the World Health Organization (WHO) had endorsed 10 global COVID-19 vaccines, including three AdV vector-based candidates. Adenovirus-based vaccine candidates, such as ChAdOx1 nCoV-19 and Ad26.COV2.S, have proven to be excellent in terms of safety and vaccine efficiency in clinical trials. This success has also led to authorization for emergency use during times of extreme need. Adenovirus vectors are gaining attention due to their advantages over traditional methods in terms of effectiveness, practicality, safety, speed of development, and commercial use [2].

Viruses can be bioengineered to remove harmful traits while being excellent at expressing therapeutic genes, creating an efficient and non-toxic gene therapy vector. However, a key challenge in most *in vivo* gene therapies is achieving effective gene delivery. Over the past three decades, viral vectors such as AdVs, adeno-associated viruses (AAVs), herpes simplex virus (HSV), lentivirus, and retrovirus have been extensively investigated for their applications in gene therapy and vaccine development. Retroviruses integrate genetic material into infected cells, but they require ongoing cell division, which is a significant limitation when used as a viral vector. Lentiviral vectors, such as human immunodeficiency virus (HIV), have a higher chance of unpredictable genome integration in the host genome. Adeno-associated virus vectors also infect both dividing and non-dividing cells with a limited immune response, but they have size constraints in terms of cargo capacity (maximum 4.7 kb). In contrast, adenovirus vectors have several distinct features, including: (1) the ability to deliver multiple copies of the transgenes into a single host cell, resulting in high gene expression; (2) transient gene expression because the DNA cargo remains episomal and does not integrate into the host genome; (3) the capability to transduce both dividing and non-dividing cells; and (4) strong immunogenicity compared to many other vectors. These advantages have been consistently demonstrated in both *in vivo* and *in vitro* studies, some of which we will discuss in upcoming sections. The primary challenge with AdV vectors is the host’s immune response when the vector is used *in vivo*. The choice of a viral vector for clinical use depends on its capacity to express foreign genes, its safety, and its stability under harsh conditions. Among these considerations, AdV vectors have emerged as highly suitable for numerous medical conditions, including difficult-to-treat cancers and vaccination against infectious diseases [3,4,5].

Adenoviruses were the first DNA viruses to undergo thorough therapeutic development due to their clear biology, genetic stability, efficient gene transfer, and easy large-scale production [6]. From 1991 to 1998, the number of clinical trials involving AdV vectors in gene therapy kept rising. However, in 1999, gene therapy as a field came to a sudden halt because of the death of Jesse Gelsinger, possibly due to a cytokine storm, who had been treated for ornithine-transcarbamylase (OTC) hepatic enzyme deficiency with AdV-based gene therapy. After Gelsinger received an injection of an AdV vector carrying a wild-type version of a defective OTC enzyme, an immediate review was prompted and raised questions on safety profile of viral vectors, more specifically of AdV vectors [7]. The patient’s unexpected death even sparked discussions on trial ethics and the essence of clinical research via numerous reports and congressional hearings. Early findings from these studies suggest that a significant factor contributing to the unfortunate incidents resulting in Gelsinger’s death is a long-standing challenge in gene therapy: the difficulty of efficiently transferring and activating genes within specific target cells [8]. During this period, the NIH addressed safety concerns associated with AdV vectors, leading to efforts to enhance their safety and efficacy. The focus shifted from single-gene therapies to broader applications, including vaccines and treatments for conditions such as cancer, arthritis, and vascular dysfunction. By 2018, AdV vectors were predominantly employed in clinical settings for cancer therapy, constituting 80% of their usage [4,9,10]. Oncolytic adenovirus vectors are engineered to replicate within tumors and naturally eliminate adjacent cancer cells. Numerous clinical trials have validated the safety and efficacy of AdV vectors, whether replicating or not. Adenoviruses have transitioned from primarily serving in gene therapy to becoming reliable vehicles for delivering vaccines. The recent 2019 pandemic has showcased the effectiveness of AdV-based vaccines in eliciting robust immune responses against viral antigens and building genuine immunity. Adenovirus-derived vectors are now widely recognized as secure and efficient carriers for vaccine components, provoking protective immune reactions against novel elements in both animal and human studies. Their potent immunogenicity makes replicating and non-replicating adenoviruses suitable for cancer vaccine development.

This review offers insights into adenovirus biology, infection, and the development of recombinant forms, including replication-defective adenoviruses and conditionally replicating adenoviruses (oncolytic vectors). It highlights their roles in modern clinical and preclinical applications, such as gene therapy, cancer treatment, regenerative medicine, and vaccination. We exclusively aim to summarize existing knowledge and recent clinical advancements, address present challenges in efficient adenovirus vector utilization, and explore future directions for their applications in cell and gene therapies, oncolytic virotherapy, and vaccination.

## 2. Biology, Species Types, Infection and Toxicity

**Figure 1:**
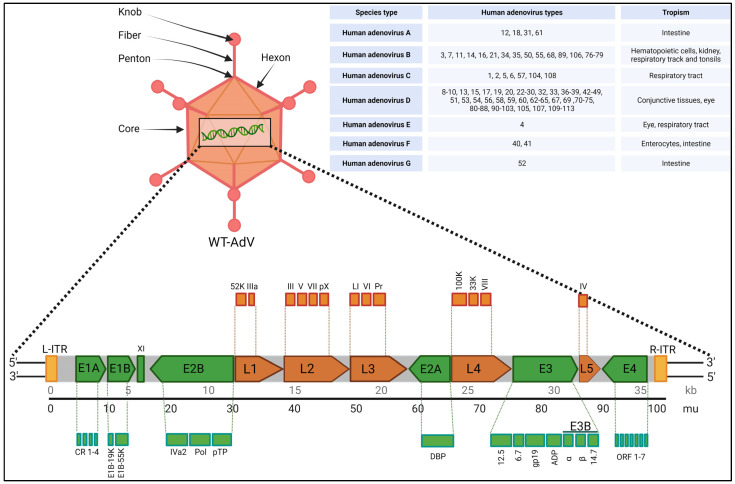
Highlights of adenovirus capsid features with overview of viral genome. In the top right corner, the species (A–G) of AdVs with their known tropism are indicated. The schematic representation of the gene map is for understanding purposes only and is not normalized for actual gene size. Kb: kilobases; mu: map unit. Created with BioRender.com (accessed on 1 December 2023).

Adenoviruses are non-enveloped DNA viruses in the *Adenoviridae* family. These viruses were first discovered by Rowe et al. in human adenoid tissues in 1953, which is why they are called ‘adenoviruses’ [11]. Adenovirus infection is typically mild for most people, but it can be very dangerous, even life-threatening, especially for immunocompromised individuals. Adenoviruses (AdVs) have a size of 80–100 nm and an icosahedral capsid. They contain linear double-stranded DNA with a genomic size ranging from 26 to 48 kb across the *Adenoviridae* family [12]. The adenovirus genome is flanked by inverted terminal repeats (ITR) [13], and it also contains a packaging signal (ψ) located on the left arm, which is crucial for viral genome packaging. The icosahedral capsid is made up of 252 capsomeres: 240 hexons (capsid protein II) forming the faces of capsid and 12 penton bases (capsid protein III) at the vertices help to cap the icosahedron’s corners. The protruding fibers (capsid protein IV) connected to each penton base help the virus attach to the host cell using its surface receptor [14]. Figure 1 highlights some of these features on the adenovirus capsid.

Adenovirus assembly happens in the following sequential order: (1) Empty capsids (procapsids) are formed with capsomers (hexons, penton bases, and fibers) and some minor capsid proteins, along with unstructured proteins. (2) The viral genome binds to packaging proteins (IVa2, L4 33K, L1 52/55K, and L4 22K) via the packaging signal within the left ITR. (3) The viral genome is enclosed within the procapsid via a unique vertex portal, leading to the release of scaffolding and some packaging proteins. (4) Precursor proteins (pIIIa, L1 52/55K, pVI, pVII, pVIII, mu, and pTP) are cleaved by the adenovirus protease (AVP), resulting in the formation of the final mature viral particle. (5) New virions are assembled within the cell nucleus two to three days after entry, leading to cell lysis and the release of virions [15,16].

Adenoviruses encompass a natural diversity of over 200 identified non-human varieties and over 100 human adenoviruses (HAdVs), which are categorized into seven species, HAdV-A to G, based on various characteristics such as DNA homology, oncogenicity, hemagglutination, and serum-neutralizing properties. However, there is ongoing debate regarding the correct classification of adenoviruses [17,18,19,20]. Differences in viral capsids delineate tropisms among the serotypes. Figure 1 lists these species and HAdVs with tissue specific tropism. Out of the different species types, ‘HAdV-D’ is the largest, with 57 known HAdVs. The primary reason for diversity in HAdV-D is gene recombination in capsid genes, such as hexons, pentons, and fibers. Interestingly, HAdV-C, while important in immunosuppressed patients, has only five types so far [21]. Children infected with adenoviruses are often affected by types 2, 1, 3, 5, 7, and 6 in that order, with types 1 and 2 accounting for around 60% of all cases. Adenovirus infections are common in humans, especially among children aged 6 months to 2 years. HAdV 2, HAdV 5, and HAdV 26 are commonly used as gene therapy viral vectors because they typically cause mild respiratory illnesses and do not lead to cancer. This makes them the preferred choice for delivering genetic material, especially for *in vivo* gene therapies, as they induce a mild immune response [22]. Most oncolytic adenovirus vectors used in virotherapy are built upon the foundation of HAdV 5, and a significant portion of AdV-based vaccines employ HAdV 5 or HAdV 26 [20].

The diverse adenovirus types offer a wide range of potential therapeutic viral vectors for clinical use after necessary genetic modifications. Figure 1 shows the tentative wild-type adenovirus (WT-HAdV) genome and the diversity of known HAdV types across seven species [20,23]; the schematic representation of the gene map is for understanding purposes only and is not normalized for actual gene size. Both early and late genes are shown on same genome considering approximate position in accordance with the HAdV 5 genome. It is around 36 kb in size and can encode more than 40 genes [24]. It has several early genes (E1–E4) and late genes (L1–L5) which in turn make many functional subunits. In addition, the adenovirus major late transcription unit (MLTU) encodes multiple proteins from five late genes, L1 to L5, via differential splicing and polyadenylation [25,26]. The early genes help turn on other viral genes and manage viral DNA replication, while the late genes (L1–L5), which encode most of the structural proteins, require the viral genome to replicate first. Briefly, after an infection, the first gene expressed is E1A, which acts as transcriptional activator and controls other genes responsible for cell and immune modulation (E1B, E3, E4 genes) and replication (E2 gene), which is crucial for activating other viral genes. Part of the E1A protein, called the conserved region 2 (CR2), pushes away retinoblastoma (pRb) and other cellular proteins, which induces the resting cells to enter the S-phase, which is a much-needed requirement for viral replication [27]. The E1B gene makes two important proteins: E1B55K and E1B19K. E1B55K takes a devious route by binding itself to the tumor suppressor p53, orchestrating its degradation via ubiquitination-driven proteasomal breakdown. This sinister collaboration serves as a strategic maneuver to thwart cell death. E1B19K also crucially prevents cell death by disrupting pathways that lead to programmed cell death, acting as an anti-apoptotic factor. E1B-19K operates upstream of mitochondria by preventing BAK-BAX assembly, effectively inhibiting apoptosis triggered via diverse death stimuli that activate the intrinsic apoptotic pathway. It also binds to BIK/NBK, disrupting the apoptotic pathway activated via protein synthesis inhibition, thereby blocking this route of cell death [28]. Together, these proteins keep the infected host cell alive and provide the ideal breeding ground for the virus to replicate prolifically [29]. Late genes create structural and core viral proteins, such as the ones making up the virus’s protective shell and its core parts. The adenovirus uses several proteins, including E1B55K, E4orf3, E4orf4, E4orf6, and core protein VII, to block the host’s DNA damage response, which would otherwise limit adenovirus replication [17,30]. Table 1 outlines the functions of some of the key genes in the adenovirus genome.

Infection stages: Our understanding of the adenovirus infection pathway predominantly relies on the knowledge derived from HAdV 5. Adenoviruses enter host cells via a process called receptor-mediated endocytosis. Adenovirus infections depend on different cell surface receptors for different species. For HAdV-C species, such as HAdV 2 and 5, the primary receptor is the coxsackie/adenovirus receptor (CAR) [31]. Attachment of host cell surface to HAdV-C species is mediated via interaction between the adenovirus fiber and the CAR receptor [32]. The secondary ligand interaction between cell surface integrins and an Arg-Gly-Asp motif (RGD motif) initiates receptor-mediated endocytosis. Once inside the cell, HAdVs go through following steps: first, they release a capsid protein called VI and escape the endosomal compartment. Next, their capsids are transported to the nuclear pore complex, where they disassemble themselves and release their genetic material into the nucleus. Once inside the nucleus, the viral genes start being expressed. However, there are two main difficulties with HAdV 5. Firstly, the coxsackievirus–adenovirus receptor needed for HAdV 5 infection is often low on cancer cells, including pancreatic cancer. Secondly, over 60% of adults have neutralizing antibodies (nAbs) that can stop HAdV 5 infection. These difficulties have pushed the scientists to look for better adenoviruses to target various cancers. For example, HAdV 35 has emerged as an alternative option for treating pancreatic cancer in a recent study [33]. Adenoviruses typically lead to respiratory problems, from the common cold to more severe conditions, such as pneumonia, croup, and bronchitis [34]. They can also cause other health issues, such as stomach infections [35], eye infections [36], bladder infections [37], and, occasionally, neurological problems [38], depending on the specific type of adenovirus.

Complete understanding of how adenovirus assembles, maintains stability, and uncoats within cells still remains a mystery. Current treatments for HAdV infections focus on interfering with viral nucleic acid metabolism, but no specific anti-adenovirus drugs are in clinical use. The virus and host share similar genetic processes, making antiviral development challenging. However, targeting the virus’s unique assembly and disassembly processes may hold promise for future drug development in treating adenovirus infections and designing anti-viral drugs [39]. Also, looking at the chromatin level, *in vitro* and *in vivo* studies by Avgousti et al. discovered a viral strategy in which nucleosome binding is exploited to control extracellular signaling. They found out that pVII forms complexes with nucleosomes, limiting DNA accessibility, and its post-translational modifications drive chromatin localization. Proteomic analysis shows that protein VII suffices to alter the host chromatin’s protein composition, retaining HMGB1, HMGB2, and HMGB3. pVII directly binds HMGB1, reducing inflammation-induced HMGB1 levels and neutrophil recruitment in mouse lungs, demonstrating its ability to sequester and prevent HMGB1 release [40]. A recent study by Schwartz et al. explores how adenovirus DNA is packaged by pVII in a chromatin-like manner and how, during early infection, host nucleosomes replace pVII at early gene sites before gene activation. Adenovirus DNA is organized like nucleosomes, and this chromatin structure undergoes remodeling during early infection. This involves de-condensation of viral gene locations and replacement of pVII with histone H3.3. Importantly, acetylation of histone H3.3 at transcription start sites precedes the activation of early genes. These studies are important because they may lead to the design of improved and more efficient HAdV vectors for gene therapy or vaccination in the near future [41].

## 3. Recombinant Adenoviruses: Safety, Modifications, Advantages, and Disadvantages

Even though many adenoviruses have been used for various medical indications and have potential benefits, there are several safety concerns. Adenoviruses can provoke the immune system very strongly because they have certain pathogen-associated molecular patterns (PAMPs) on the virus’ outer shell, DNA, and some intermediates parts. When the immune system detects these patterns, it might release cytokines and chemokines that can cause excessive inflammation, a process called a cytokine storm. Additionally, because many people have encountered AdVs before, their immune systems may have developed defenses, such as nAbs, which could stop the virus from working as efficiently as intended. To address safety concerns and increase gene payload capacity, newer versions of adenoviruses have been designed over the years. These improvements reduce harmful reactions, eliminate replication-related genes, and allow for more genetic material to be carried. Recombinant adenoviruses (rAdVs) are modified viruses created in laboratories by removing viral genes/adding foreign genes of interest. In gene therapy and vaccines, mostly replication-defective rAdV viral vectors are used to efficiently deliver the inserted genetic material into cells, enabling the expression of desired proteins or triggering specific immune responses, but rAdV vectors in oncolytic virotherapy are modified based on following two principal strategies:

In the first strategy, tumor cells are transduced with therapeutic transgenes carried by replication-defective rAdVs which produces anti-cancer affects. The key advantages include increased expression of the therapeutic gene within the tumor cell, a better safety profile, and reduced expression of viral proteins. Another benefit is that these rAdV vectors can be concentrated to a very high level of vector particles per milliliter (vp/mL), and they can infect both dividing and non-dividing cells [42].

In the second strategy, selectively replication-competent lytic viruses are engineered to replicate exclusively within tumor cells, resulting in the destruction of cancerous cells via lysis. This amplifies the therapeutic gene within the tumor cell and facilitates the spread of transgenes throughout the entire tumor [43]. Various genome engineering methods have been undertaken to modify AdVs to unlock their full potential, but there are still limitations when using rAdVs as vectors. For example, as discussed earlier, Species C (HAdV 5) vectors naturally target certain receptors such as coxsackie/adenovirus receptors (CAR). This in turn hinders cell-specific targeting by HAdVs. Additionally, these tumor target cells have few surface receptors, leading to weak infections and susceptibility to immune responses in the *in vivo* gene therapy. There are currently four main types of rAdV constructs that have been engineered to fit diverse therapeutic applications:

(i) First-generation adenovirus (FGAdV) vectors: These vectors remove the E1 and E3 regions, allowing for increased cargo capacity by eliminating non-essential genetic information. These vectors cannot replicate on their own and rely on packaging cells, such as human embryonic kidney 293 cells expressing E1 protein, for production. The vector is capable of carrying up to 8.2 kb of foreign DNA cargo with higher immunogenicity [44]. These were used in certain earlier gene therapies. Vaccines often use FGAdV to trigger an apt immune response Modifications of early genes mostly depends on specific application.
(ii) Conditionally replicating adenovirus (C-RAdV) vectors: These modified viral vectors involve the removal of E1 and/or E2 units that make it replication-deficient [45]. The vector is capable of replicating selectively in tumor cells, but not in normal cells, due to their abnormal retinoblastoma protein [46]. The engineering of tumor-specific gene promoters into the AdV genome is used to control the induction of viral replication to construct C-RAvD, which is regularly used in oncolytic virotherapy.
(iii) Second-generation adenovirus (SGAdV) vectors: This generation of adenovirus was created to hold more genetic material (up to 12 Kb). The FGAdVs, despite E1 region deletion, induce strong host immune responses due to E1A-like factors in human cells. The SGAdV with E2 and E4 deletions was created to address this, but it still triggers host immune responses, resulting in reduced transgene expression in target cells [47]. More gene deletion was created by removing genes such as E2 and/or E4, in combination with E1 and E4 gene deletions. These are used in certain gene therapies for genetic disorders, vaccines, and certain types of cancers.
(iv) Third-generation adenovirus (TGAdV) vectors: FGAdV and SGAdV vectors, despite E1–E4 region deletion, exhibit substantial immunogenicity and cytotoxicity. To accommodate larger therapeutic genes, third-generation adenovirus vectors were developed by removing the entire native adenovirus genome except for essential elements, thus raising the cargo limit to 36 kb. These ‘gutless adenovirus vectors’ enable high-level gene expression with minimal immune response. Production requires a co-introduced ‘helper adenovirus,’ but contamination concerns have led to the development of contamination-free methods using plasmids as helpers [48]. Incorporating stuffer DNA for efficient encapsidation in third-generation vectors has variable effects on transduction efficiency, with conflicting reports in the literature. These vectors do not have many regulatory genes and also do not have genetic elements, such as packing signals [48,49]. These gutless and safer vectors are regularly used in certain gene therapies for genetic disorders, vaccines, and certain types of cancers.

**Figure 2:**
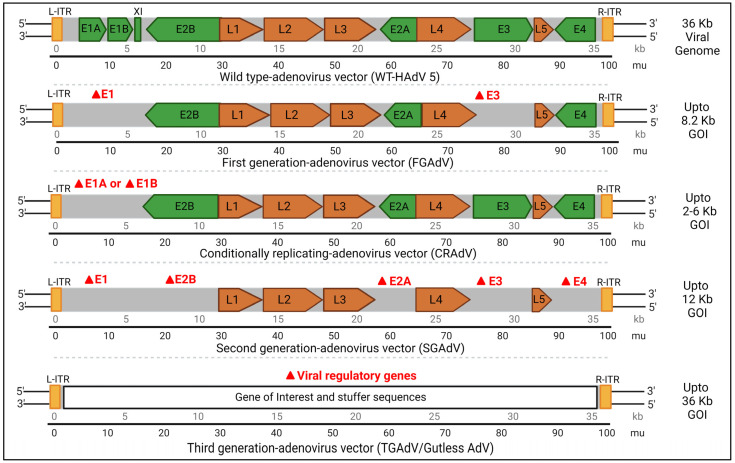
Represents an overview of the scope of the modifications and packaging capacity of rAdV vectors. Red delta signifies the possibility of gene deletion for creating multi-generational rAdVs. The suggested approximate insert size of the gene of interest (GOI) depends on the specific application. Early and late genes are explained in previous sections. The schematic representation of the gene map is for understanding purposes only and is not normalized for actual gene size. Kb: kilobases; mu: map unit Created with BioRender.com (accessed on 1 December 2023).

Figure 2 shows the possible modifications to and packaging capacities of rAdV vectors. The schematic representation of the gene map is for understanding purposes only and is not normalized for actual gene size. Modifications are indicated as the last stage resultant vector. Currently, more than 20% of gene therapy trials for cancer use HAdV 5 as a delivery vector, and two gene therapy drugs packaged with HAdV 5 (Gendicine and Oncorine) are approved for use in China [47].

## 4. Recombinant Adenovirus Vector Production Methods

The generation of rAdV vectors typically involves techniques such as straightforward cloning recombination or using *Escherichia coli* (*E. coli*) cells for genome engineering [2]. Numerous approaches are discussed as below:

(i) The traditional method/homologous recombination: The classical method for obtaining E1-deleted rAdVs involves homologous recombination of two DNA vectors. One vector contains a sequence mapping to the gene of interest at the left end of the adenovirus genome, while the other carries a sequence overlapping the 3′ viral segment and extending to the adenovirus genome’s right ITRs. This method is mostly used for generating FGAdV. This recombination process takes place in E-1-expressing cells such as HEK-293 cells. However, this laborious method is very inefficient in terms of recombination events and time consumption [50].
(ii) Cre-LoxP-mediated recombination: To overcome limitations of the classical method, a Cre-lox site-specific recombination approach was developed. It involves three components: (a) a recombinant adenovirus with two loxP sites; (b) a shuttle vector containing ITR, an expression cassette, a packaging signal, and a loxP site; and (c) a 293-Cre cell line expressing Cre-recombinase. Transfection of the shuttle vector, containing the gene of interest and viral DNA, into a 293-Cre cell creates an adenovirus genome capable of replication, but it cannot be packaged. Recombination occurs between the loxP sites of the generated adenovirus genome and the shuttle vector, yielding the desired recombinant adenovirus. One drawback is the presence of parental adenovirus in the preparation, which persists even after multiple passages in 293-Cre cells, requiring careful recombinant virus verification [47,51].
(iii) The AdEasy approach: This method employs HEK 293 cells to minimize homologous recombination issues, leveraging recombination in microorganisms such as yeast and bacterial cells. For instance, the AdEasy system aids in recovering the recombinant *E. coli* clone, primarily by introducing expression cassettes into the E1 region. After purifying recombinant plasmid DNA, it releases the viral chromosome and is subsequently transfected into the cell line. This system predominantly relies on *E. coli* rather than mammalian cells, benefiting from the bacterial machinery’s homologous capabilities [2,47].
(iv) Use of Helper AdV for making TGAdV: The genome size of a virion should be within the range of 27.7–37 kb for proper packaging [52]. As discussed earlier third generational AdV/gutless AdV are helper-dependent adenovirus vector. TGAdV genomes include a noncoding eukaryotic ‘stuffer’, adenovirus ITRs, and a packaging signal [53]. In contrast, the helper virus (HV) lacks E1 and contains a packaging signal flanked by loxP sites. Infecting 293-Cre cells allows for the removal of the packaging signal from the helper virus genome, making it unpackageable but still capable of DNA replication, complementing TGAdV genome replication and encapsidation. An alternative TGAdV production system based on FLP/FRT site-specific recombination has also been developed, with similar results [54]. During packaging, helper virus contamination can be reduced via the Cre/LoxP or FLP/FRT systems if necessary [2,47]. Guo et al. recently conducted a study on restriction assembly for making a novel adenovirus vector. This is an easy-to-use method without the need for sophisticated instruments [55].

## 5. Applications of Recombinant Adenovirus Vector in Gene Therapy, Cancer, and Regenerative Medicine

An essential part of developing gene therapy viral vectors is selecting the best way to deliver transgenes effectively. Recombinant AdVs have packaging capacity that is up to seven times greater than that of rAAVs and have had great success in basic research and clinical applications. We will discuss in the following sections some of these important preclinical and some ongoing clinical trials which have been conducted in gene therapy, virotherapy and vaccines using rAdV vectors. Table 2 lists a selection of active clinical trials using rAdVs as vectors (data accessed from clinicaltrials.gov (accessed on 20 October 2023) and previously published articles) [56,57].

### 5.1. Recombinant Adenovirus Vector Applied in Gene Therapy

**Figure 3:**
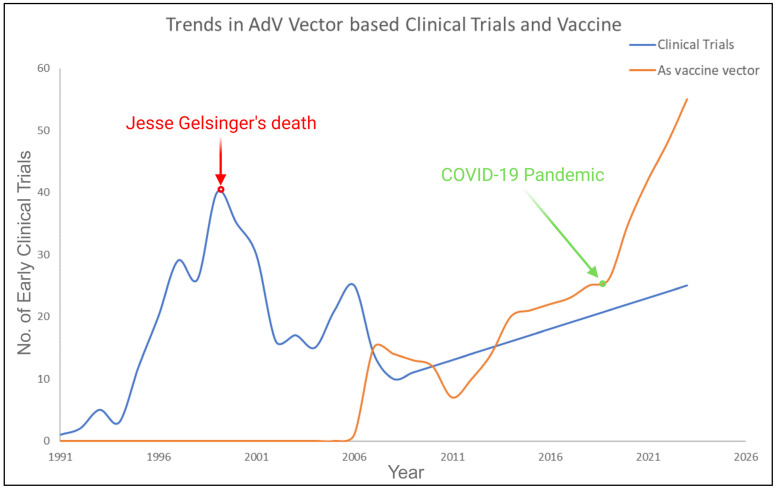
Shows an overview of the clinical trials trend. The red highlighted point on the graph indicates the decline in adenovirus vector application and the green highlighted section represents the recent pandemic, which points towards the upward trajectory of revamped interest in adenovirus vectors. Created with BioRender.com (accessed on 1 December 2023).

In 1991, Rosenfeld et al. conducted the first *in vivo* gene transfer using rAdV vectors, showing that they could deliver the human α 1-antitrypsin (A1AT) gene to a cotton rat’s lung [58]. In 1993, the first human gene therapy study utilizing rAdV vectors was conducted. A 23-year-old patient with cystic fibrosis became the recipient of the inaugural *in vivo* gene therapy, featuring an E1-E3-deleted rAdV delivering the normal hCFTR gene. This led to further clinical studies employing rAdV vectors. Regrettably, multiple studies afterwards revealed that rAdVs are highly immunogenic. This not only impeded targeted transgene delivery and specific cell gene expression but also triggered unwanted immune responses in treated individuals. In 1999, the death of Jesse Gelsinger during a clinical trial using an adenovirus vector for OTC gene therapy raised significant safety concerns and caused a decline in related studies for the next decade. It was found that the capsid protein’s innate response triggered a cytokine storm. These unfortunate findings and supporting evidence in animal models indicated that even with gutless rAdV designs (TGAdV), the capsid could still provoke a potent immune response, limiting the use of rAdV vectors in human gene therapy. Figure 3 shows an overview of the trend of rAdV vectors in clinical trials for gene therapy (blue) and vaccine (orange) delivery across different diseases from 1991 to 2023, based on stipulations from previous prestigious articles and clinical trial.gov site (accessed on 20 October 2023) [10,47,59,60].

The landscape of research during that time witnessed a significant shift towards the development of viral vectors with reduced immunogenicity, ultimately giving rise to the increased prominence and utility of recombinant AAV and recombinant lentiviral vectors in the gene therapy field. Recombinant adenovirus has emerged as a promising candidate for both oncolytic virus therapy and vaccine delivery due to its tendency to induce immune response, as illustrated in the compelling trends depicted in Figure 3. Sheldon et al. recently analyzed the safety of HAdV 5 as a delivery vector in various oncologic clinical trials and specific drug route-delivery options. Their research showed that it is generally safe, suggesting a need to reconsider it as a carrier for gene therapy products [61]. Now, adenovirus-based gene therapy trials represent 50% of global clinical trials, primarily focusing on innovative vaccines and cancer treatments [57]. This is estimated to increase even more by 2024, driven by additional successes at the clinical stage and commercial approvals in the market.

### 5.2. Recombinant Adenovirus Vector Applied in Oncolytic Virotherapy

Cancer is considered a life-ending malady in most cases. The reason behind this challenge is that many cancers become incurable once they metastasize. Cancer treatment typically relies on surgery, radiation, and anticancer drugs, with immunotherapy becoming a standard option, as applicable depending on the type of cancer. However, surgery can be taxing and is not always suitable for each type. Conventional treatments such as chemotherapy and radiotherapy often come with significant life-debilitating side effects. This is partly due to the adaptability of tumor cells during treatment, often resulting in resistance and becoming repetitive treatment loops. Oncolytic virus therapy, also known as oncolytic virotherapy, on the other hand, is gaining attention, as it minimally impacts patients’ well-being. Oncolytic viruses primarily infect and replicate within cancer cells, leading to the cells’ destruction. The released viruses can then infect nearby cancerous cells, creating a cascading effect within tumor tissues. In clinical trials, HAdV 5 is utilized to carry out oncologic gene therapies that work in different ways. These therapies can be grouped into three main categories:

1. Suicide genes: These genes make an enzyme that, when given a prodrug, triggers cell death. Suicide gene therapy approach is mostly used for solid tumors. After adenovirus is injected into the tumor (ITU), inactive prodrugs can be broken down into cytotoxic metabolites, leading to cell death. Adenovirus vectors have been designed to activate the p53 pathway, causing cell-cycle arrest and apoptosis in tumor cells, as many tumor types exhibit p53 dysfunction [62,63]. However, not all cancer cells lack p53. Various applications of adenoviruses in anticancer therapy have been explored beyond targeting p53 dysfunction. For example, herpes simplex virus thymidine kinase (HSV-TK) can convert prodrugs into cytotoxic compounds, such as converting fludarabine monophosphate (F-ara-AMP) into fluoroadenine or ganciclovir (GCV) into a cytotoxic nucleotide. It inhibits DNA polymerase and/or leads to incorporation into DNA and causes chain termination and tumoral cell death [64]. Additionally, the enzyme cytosine deaminase (CD) converts the prodrug 5-fluorocytosine (5-FC) into cytotoxic 5-fluorouridine (5-FU), causing DNA damage [65]. Other approaches include using varicella zoster virus-thymidine kinase (VZV-tk), purine nucleoside phosphorylase (PNP), and nitroreductase (NR). Modified adenovirus vectors delivering CD/HSV-TK chimeric enzymes, often in combination with other therapies such as radiotherapy, have shown effectiveness in clinical trials for various cancers, including prostate and pancreatic cancer [66,67]. Some vectors also carry additional genes, such as the human sodium iodide transporter (hNIS) for tumor imaging or pro-inflammatory cytokines such as IL-12 to trigger antitumor immune responses [68].
2. Immunostimulatory genes: They introduce genes that regulate the immune system into the tumor cells, leading to a focused immune response. Adenovirus vectors can be equipped with immune-boosting genes to trigger the patient’s immune system against tumors. Delivering interferon (IFN)-β or IFN-α-2b using AdV vectors directly into the lungs has been shown to be a safe therapy for malignant pleural mesothelioma [69]. In Table 2, there are multiple active clinical trials ongoing using this strategy.
3. Tumor suppressor drugs: These therapies reactivate the mutated tumor suppressor pathway. Tumor suppressor genes, such as p53, p16, RB, PTEN, and WWOX, are critical for controlling cell growth and differentiation under normal circumstances. Various strategies for tumor suppressor gene therapy exist. One such therapy, Gendicine, has been approved for clinical use. Combining p53 with treatments such as radiotherapy and chemotherapy has shown promise in treating cervical and liver cancers. Another approach involves inhibiting MDM2, a negative regulator of p53, with drugs such as RG7112, showing clinical activity in leukemia treatment [70]. Converting mutant p53 into a functional form using metallochaperones is another strategy, while vaccines targeting p53 mutant proteins have shown success in preclinical trials [71,72]. The adenovirus-mediated pRb94 has inhibited lung cancer cell growth, but wild-type Rb gene activation is challenging [73]. Additionally, introducing the PTEN gene can enhance the antitumor effect and reduce drug resistance in ovarian cancer cells. It has been evaluated with the rAdV approach as well [74].

Now let us dive into past and more recent trials regarding virotherapy using the above discussed therapeutic approaches.

The first oncolytic virus, ONYX-015 aka DL1520, was developed as a defective AdV with deletion in E1B55k subunit [75,76]. It selectively targeted and destroyed human cancer cells with non-functional p53 genes. Clinical trials for head and neck cancer treatment were conducted, but ONYX-015 had to be directly injected into tumors due to its high intravenous toxicity, limiting its use to large tumors only. Unfortunately, it did not deliver the expected antitumor results, leading to the project’s termination in 2000 [77]. However, a modified version called H101 (Oncorine) gained approval from the Chinese Food and Drug Administration in 2005 for head and neck cancer treatment, as well as Rigvir, which has uncertain efficacy and is approved in Latvia [78,79,80]. To further increase cancer cell infectivity and specificity in AdV vectors, modifications are carried out by swapping the HAdV 5 fiber knob with different adenovirus serotypes for changing tropism as well. Initially, researchers incorporated the HAdV 35 fiber knob into HAdV 5, and ongoing research is exploring the use of HAdV 3 and HAdV 37 fiber knobs with the same approach [81]. The candidate, H101, shows antitumor effect, and the latest clinical trial on H101 (NCT05124002) is aiming to further the effects and safety of this vector when combined with hepatic artery infusion chemotherapy of FOLFOX regimen for intrahepatic cholangiocarcinoma. An ongoing Phase I trial (NCT05717712) will look for possible hematologic and neurologic toxicity of AdV-TD-ncIL12 to determine the maximum tolerated dose of modified oncolytic rAdV 5. This new rAdV 5-based AdV-TD variant has three gene deletions (E1ACR2, E1B19K, and E3gp19K), while it retains the E3B gene. This mutant oncolytic adenovirus demonstrated potent *in vivo* antitumor effects. Building upon AdV-TD, AdV-TD-nsIL12 is equipped with human non-secretory interleukin-12 [82]. AdAPT-001 is undergoing a clinical trial (NCT04673942) for potential treatment of refractory solid tumors involving a replicative type 5 adenovirus equipped with a fusion trap of TGF-β receptor and immunoglobulin Fc. Its purpose is to counteract isoforms 1 and 3 of TGF-β, which are known for their pro-fibrotic and immunosuppressive properties [83]. At present, a Phase III clinical trial (NCT04452591) is actively underway. This trial is focused on the treatment of non-muscle-invasive bladder cancer. The experimental treatment involves the intravesical administration of n-dodecyl-β-D-maltoside (DDM) in combination with a bioengineered oncolytic adenovirus (CG0070). Numerous ongoing clinical trials (most relevant and active) are documented in Table 2.

### 5.3. Recombinant Adenovirus Vector Applied in Regenerative Medicine and Stem Cell Related Research

Regenerative medicine based on genetic engineering and human pluripotent stem cells (HPSCs) is a potential innovative therapy for multiple fatal diseases. The application of rAdV has been studied by very few researchers in this aspect [30]. Initially, rAdV-based gene therapy was applied for bone tissue engineering to augment growth factors using common AdV vectors. Recombinant AdV vectors expressing osteoinductive growth factors were aimed to induce osteogenic differentiation, ossification, and integration with surrounding bone tissue. This can be achieved using osteoinductive growth factors in both *in vivo* and *in vitro* settings. In 1999, Lieberman et al. compared rhBMP-2 and BMP-2 delivered via adenovirus vector gene transfer in rat skeletal defect healing. Gene transfer showed superior results, with increased bone formation quality and quantity [84]. Subsequent studies further supported gene therapy for enhancing bone engineering with BMP [85]. Gene therapy techniques were initially established for studying bone engineering and were subsequently refined [86]. Various factors in the bone formation process were investigated, including mesenchymal stem cell sources, injection sites, and *in vivo* vs. *ex vivo* approaches. These efforts played a significant role in advancing bone engineering, leading to clinical trials and FDA approval for BMP-2 and BMP-7 in spinal fusion and fracture repair. Although not in clinical use yet, BMP-9 has shown strong osteoinductive potential in preclinical models, suggesting potential future clinical applications [47]. Adenovirus gene delivery has been utilized in various preclinical studies within regenerative medicine and tissue engineering. Examples include improved intervertebral disc healing in rabbits via Sox9 expression [87], effective transgene delivery to rabbit flexor tendons [88], and accelerated tendon healing with increased strength in rats via AdV-mediated BMP14 transfer [89]. These studies observed no adverse immunological responses or unwanted bone or cartilage formation in host tissues.

In stem cell-related research, Chen et al. have successfully achieved targeted delivery to treat malignant brain tumors by using perfusion-guided infusion of mesenchymal stem cells loaded with tumor-selective oncolytic rAdV, DNX-2401, Delta-24 (MSC-D24). Presently, a clinical trial is underway utilizing a similar approach (NCT03896568) [90]. Wang et al. developed a non-invasive adenovirus-based gene therapy method for hematopoietic stem cells (HSCs) involving IV injections, eliminating the need for intensive treatments and transplantations. They identified a new receptor called desmoglein 2 (DSG2) for *in vivo* HSC transduction. Using specialized HAdV 5/3+ vectors, they achieved higher marking rates in HSCs, making it a viable option for gene editing therapies. This study also sheds light on HSC behavior after mobilization in non-human primates [91]. A recent study by Haruta et al. investigated adenovirus reactivation after stem cell transplantation, a potentially life-threatening condition. It compares the commonly used qPCR method to a more precise one called droplet digital PCR (ddPCR) for detecting adenovirus DNA. Analytical ddPCR demonstrated higher sensitivity and reproducibility, particularly in pediatric patients. This improved accuracy can aid in setting treatment criteria and evaluating antiviral therapies [92]. Ongoing clinical trials are investigating the optimal dosage and potential adverse effects of an autologous dendritic cell-adenovirus CCL21 vaccine (CCL21-gene modified dendritic cell vaccine) when used in conjunction with intravenous pembrolizumab. The aim is to assess their efficacy in the treatment of stage IV non-small cell lung cancer patients (NCT03546361).

## 6. Vaccine Related Recombinant Adenovirus Development

Recombinant adenovirus vectors can deliver foreign epitopes to boost the host’s immune response against pathogens, promoting the production of pro-inflammatory cytokines and effective adaptive immune responses [22]. These advancements have established rAdV vectors as excellent vaccine carriers. In the 1970s, the US Army created live AdV vaccines to prevent respiratory diseases caused by HAdV 4 and HAdV 7 infections [93]. During the period from 1980 to 1999, scientists worked on modifying vectors by removing their replicative early genes and inserting them into specific cell lines, allowing these viruses to replicate in those cells (different generations of AdV vectors). Unfortunately, Jesse Gelsinger’s tragic death during OTC gene therapy resulted from being inoculated with an astounding 38 trillion viral particles, triggering a cytokine storm. This incident re-prompted the exploration of rAdV vectors in vaccine development. One major benefit of using rAdV vectors in vaccines is the elimination of additional adjuvants. The AdV vector itself is sufficient to induce controlled inflammation when administered in low doses. In 2014, the Chinese company CanSino Biologics developed an AdV-based vaccine during an Ebola outbreak, although its approval was limited to emergency use only [94]. These results encouraged scientists to explore recombinant AdVs as potential vehicles for developing COVID-19 vaccines during the recent pandemic. Let us explore more into latest clinical advancements in vaccine development, starting with viral diseases (in alphabetic order), followed by a brief overview of bacterial and parasitic conditions and their respective vaccine developments.

Ebola Virus Disease: Ebola virus disease (EVD) is a rare, severe, often fatal illness in humans caused by filovirus Ebola virus. It is transmitted from animals to humans and then spreads among the population. The average fatality rate is approximately 50% and causes gastrointestinal manifestations and multiple organ dysfunction syndrome [95]. Effective Ebola virus vaccination aims for both short- and long-term immunity in patients. Ebola vaccines using recombinant AdV vectors triggered specific antibodies and T cell responses in multiple clinical trials [96,97,98,99]. In a Phase III interventional trial (NCT04152486), a two-dose Ebola vaccine (HAdV26.ZEBOV, MVA-BN-Filo) is planned to be tested in the Democratic Republic of the Congo [100]. An Ebola vaccine trial on 800 participants with HAdV.26-ZEBOVAC/MVA-BN-Filo, in particular, was safe and led to long-lasting immune responses lasting over a year after vaccination (NCT04028349). The positive outcomes of clinical trials for the Ebola vaccines such as ChAdV3-EBO-Z and HAdV 26.ZEBOV have reignited interest in HAdV 26 [101,102,103]. HAdV 26.ZEBOV/MVA-BN-Filo received regulatory approval in the European Union in 2021 [104]. A currently ongoing Phase II clinical trial (NCT06036602) will evaluate safety and tolerance in healthy adults with ChAdV 3 based vaccine. The recombinant chimpanzee AdV 3-vectored Sudan Ebola virus vaccine, (ChAdV 3-EBO S), is composed of a ChAdV 3 vector that expresses wild-type glycoprotein (WT GP) from the Sudan Gulu Ebola virus strain. Marburg hemorrhagic fever is a severe and often deadly illness caused by a virus belonging to the same family as the one responsible for Ebola hemorrhagic fever. While both diseases are infrequent, they can lead to significant outbreaks with a high mortality rate. Presently, there are no targeted treatments or vaccines available for these conditions. An ongoing Phase II clinical trial (NCT05817422) is trying to evaluate safety and tolerability of a monovalent chimpanzee ChAdV 3 vaccine expressing Marburg wild-type glycoprotein (WT GP) from the Angola strain.

Genital Herpes (GH): Genital herpes is now prevalent worldwide, especially in developing countries. The HSV-2 virus is responsible for about 90% of cases. Therapeutic HSV-2 vaccines aim to help those already infected by reducing outbreaks and virus shedding. Wan et al. assessed three rAdV-based vaccines (rAd-gD2ΔUL25, rAdV-ΔUL25, and rAdV-gD2) for their therapeutic potential against acute and recurrent diseases in guinea pigs. Results showed that rAdV-gD2ΔUL25 induced higher binding antibody levels, while rAdV-gD2 + rAdV-ΔUL25 led to higher neutralizing antibody levels compared to controls. Both rAdV-gD2ΔUL25 and rAdV-gD2 + rAdV-ΔUL25 significantly improved survival rates by 50% and reduced viral replication in the genital tract and recurrent genital skin disease compared to rAdV-gD2 [105]. These findings offer fresh insights into HSV-2 therapeutic vaccine development and introduce a potential technique to mitigate the growing spread of HSV-2.

Acquired immunodeficiency syndrome (AIDS): Human immunodeficiency virus (HIV) is a virus that weakens the body’s immune system and in turn causes acquired immunodeficiency syndrome (AIDS), making it easier for a person to become sick from other, simpler infections and diseases. It spreads via contact with specific bodily fluids, typically during unprotected sex or when sharing dirty needles. Despite almost 40 years of research, we are still working on finding a safe and effective HIV vaccine. The challenge lies in the virus’s genetic diversity and its clever ways of evading the immune system. The immune system struggles to recognize HIV envelope glycoproteins, which makes it rare to produce strong protective antibodies either during the infection or via vaccination [106]. Recombinant AdV-based vaccines for HIV, which each focus on specific HIV-1 genes such as gal, pol, and env (HAdV 5-gag, HAdV 5-pol, and HAdV 5-Nef), have been created and tested. Unfortunately, these trials did not demonstrate effective vaccination [107,108,109,110]. Consequently, in the early 2000s, major vaccine players began employing rAdVs as a delivery vehicle. However, the initial attempt to create an HIV vaccine using rAdV vectors in 2007 did not yield the desired outcomes, leading to a hiatus in rAdV usage for vaccine development for five years [107,111]. The initial assessment in humans of a vaccine based on a recombinant HAdV-D26 virus expressing the HIV-1 envelope protein (Env) from clade A revealed that this vector prompted a wide range of specific immune responses in both humoral and cellular aspects. A single IM administration of the HAdV-D26-vectored HIV-1 Env vaccine triggered immune responses both in the body’s general circulation and the mucosal surfaces. Importantly, the development of specific immune responses in the mucosal system did not appear to cause noticeable inflammation in these areas [109,112]. A recently completed Phase III clinical trial (NCT03964415) utilized AdV 26-based vector on 3900 participants. The AdV26-Mos4-HIV vector together with Clade C and Mosaic gp140 HIV bivalent vaccine was injected intramuscularly into the deltoid muscle. The Ad26.Mos4.HIV is a tetravalent vaccine which is composed of AdV26.Mos1.Gag-Pol, AdV26.Mos2.Gag-Pol, AdV26.Mos1.Env, and AdV26.Mos2S.Env. The Clade C and Mosaic gp140 HIV bivalent vaccine contains Clade C gp140, HIV-1 Env gp140 of Clade C, Mosaic gp140, HIV-1 Env gp140, and aluminum phosphate adjuvant. The outcome of vaccine efficiency of these heterologous vaccine regimen is expected by the end of 2023. An ongoing Phase I clinical trial (NCT03878121) will evaluate safety and immunogenicity of AdV 4-based HIV envelope protein-expressing vaccine. The vaccine recipients will receive an intranasal spray of either AdV4-Env150KN or AdV4-Env145NFL, containing 5 × 10^8^ viral particles at months 0 and 2. At month 6, all vaccine recipients will receive an intramuscular protein booster vaccination with the soluble trimeric protein VRC-HIVRGP096-00-VP (Trimer 4571) along with alum. Immunogenicity assessments will be conducted at baseline and specified time points up to month 12. However, as of September 2023, there is no AdV-based HIV vaccine that has proven successful.

Human papilloma virus infection (HPV): HPV causes genital warts, which lead to painless growths or lumps around the genitals. Some HPVs, such as HPV 16/18, are considered very high risk and may progress to cause cervical dysplasia, which may progress to cervical cancer. HAdV-D26 vectors expressing HPV16 and HPV18 antigens induced specific CD8^+^ T cell responses in mice, including the cervicovaginal tract [113]. This suggests the potential for therapeutic vaccination against persistent HPV infection and cervical intraepithelial neoplasia, especially when considering various disease stages.

Flu (Influenza): The flu is a contagious respiratory sickness caused by influenza viruses that infect the nose, the throat, and occasionally the lungs. Symptoms can range from mild to severe illness and, in some cases, lead to death. Clinical trials have assessed human AdV-based influenza vaccines capable of producing key influenza viral antigens such as HA, NP, and M2, including trials for H1N1 and H5N1 (NCT03232567 and NCT00755703). Additionally, a chimpanzee AdV vector called ChAdOx1, expressing NP and M1 antigens, was developed and tested in two Phase I trials (NCT01818362 and NCT01623518) [57]. The University of Oxford-developed ChAdOx1 vector with NP + M1 has demonstrated an excellent safety profile in the influenza trial FLU004. A Phase II clinical trial (NCT02918006) completed in 2022 using AdV vector has also shown great promise to be advanced to Phase III. Human Influenza A (H1N1) challenge following administration of an oral H1N1 HAdV-vector based seasonal influenza vaccine and dsRNA adjuvant to healthy adult volunteers’ results proved to be good [114]. The orally administered vaccine was well tolerated and generated protective immunity against virus shedding, similar to a licensed intramuscular IIV. These results represent a major step forward in developing a safe and effective oral influenza vaccine [115].

Middle East Respiratory Syndrome (MERS): Middle East Respiratory Syndrome is a contagious respiratory illness that can be very deadly and is caused by MERS-CoV virus. It spreads very swiftly via close contact with an infected person and causes symptoms such as fever, cough, shortness of breath, and sometimes nausea, vomiting, and diarrhea [116]. The first clinical study (NCT03399578) on MERS carried out by the University of Oxford developed a proven vector, ChAdOx1. ChAdOx1-MERS vector exhibited favorable safety and tolerability profiles across all dosage levels. Even a single dose demonstrated the capacity to stimulate both humoral and cellular immune responses against MERS-CoV vector. The outcomes of this initial human clinical trial provided a solid foundation for advancing clinical development into the subsequent phases of field trials [117]. Recently, Phase Ib (NCT04170829) was finished in 2020. The outcomes were very positive and combined with immunogenicity data from a trial in the UK, ChAdOx1-MERS vaccine has proven to be good option for advancing into Phase II clinical evaluation [118].

Stomach bug infection (Norovirus infection): Norovirus, sometimes referred to as the winter vomiting disease, is the most common cause of gastroenteritis. Infection is characterized by non-bloody diarrhea, vomiting, and stomach pain. This is a very contagious virus which spreads via food or water that is contaminated during preparation or via contaminated surfaces. In terms of recent clinical work, NCT05212168 has been going on since last year. This Phase IIb trial is using E1/E3 deleted HAdV 5 virus with dsRNA adjuvant. The vaccine vector encodes a full-length VP1 gene from Norwalk virus (NV). The adjuvant attached is a short hairpin RNA with 21 base tandem repeats flanking six bases in the center. This study hypothesize that Norovirus vaccine (VXA-G1.1-NN) will protect against gastroenteritis related to norovirus infection in the challenge mode. Estimated study completion date is around June 2024. Upon success of this vaccine, more players are anticipated to enter in clinical phases.

Respiratory syncytial virus infection (RSV): The respiratory syncytial virus causes nearly 64 million acute respiratory infections worldwide annually in older adults and in persons with underlying chronic cardiac or pulmonary conditions [119,120]. The safety and immunogenicity of AdV26.RSV.preF was accessed in Phase I/IIa (NCT03303625) which successfully demonstrated immunogenicity in healthy adults and toddlers with no safety concerns raised. A recently completed Phase IIb clinical trial (NCT03982199) by Janssen showed promising results and confirmed AdV26.RSV.preF-RSV preF protein vaccine was immunogenic and prevented RSV-mediated lower respiratory tract disease [121]. Overall, 5782 participants were enrolled and vaccine vs. placebo efficiency was found to be 80%, with 2791 received vaccine [121]. Evaluations regarding RSV-seronegative children are currently underway [122]. Normally, RSV and influenza infections overlap in seasons. Sadoff et al. conducted a study involving older adults (NCT03339713), co-administering the AdV26.RSV.preF investigational vaccine with a seasonal influenza vaccine (Fluarix). There was no evidence of immune response interference, and it was found to be safe. This suggests that simultaneous seasonal vaccination with both vaccines is compatible [123]. There are currently two RSV vaccines approved for adults ages 60 years and older—RSVPreF3 (Arexvy, GSK, Brentford, UK) and RSVPreF (Abrysvo, Pfizer, New York, NY, USA). In May 2023, the US FDA approved the Arexvy vaccine for adults aged 60 years and older to be protected from RSV disease. The green light stems from compelling results in the pivotal AReSVi-006 Phase III trial, demonstrating remarkable efficacy in older adults, including those with underlying health issues and severe RSV disease. The U.S. launch is scheduled ahead of the 2023/24 RSV season [124]. In August 2023, the US FDA approved RSVpreF vaccine (Abrysvo, Pfizer, New York, NY, USA) for pregnant persons as a single dose to prevent RSV-associated lower respiratory tract disease [125,126]. Adenovirus-based RSV vaccine awaits market entry; further research needed for enhanced efficacy and efficiency.

Rift valley fever (RVF): Rift valley fever (RVF) is a mosquito-transmitted disease caused by Rift Valley fever virus (RVFV) that causes severe disease which ranges from a mild flu-like illness to severe hemorrhagic fever, in animals and humans. Preclinical work was carried out by Hao et al. utilizing rAdV vectored RVF vaccine and confirmed that vaccine presents comprehensive protection against lethal RVFV challenge in A129 mice. Intramuscular HAdV 5-GnGcopt immunization in mice generated durable antibodies and strong cellular immune responses. A single HAdV 5-GnGcopt vaccination fully protected interferon-α/β receptor-deficient A129 mice from lethal RVFV infection. HAdV5-GnGcopt shows promise as an RVFV vaccine candidate, but further research is required to verify its efficacy in a natural animal host and assess its potential as a human vaccine candidate [127]. Nevertheless, there are no vaccines to prevent RVF infection for human at present which have been licensed or made commercially available.

COVID-19 (SARS-CoV-2 virus infection): The COVID-19 pandemic, caused by SARS-CoV-2, is a severe global health crisis with over 770 million infections and 6 million deaths worldwide [128]. A total of over 13 billion vaccine doses have been administered [WHO Coronavirus (COVID-19) Dashboard accessed on 11 September 2023]. Already-approved vaccines and current developmental candidates are primarily designed to target the spike (S) protein of SARS-CoV-2, which is the prominent antigenic feature of the virus. Table 3 lists clinically approved AdV-based vaccines for COVID-19 and other therapies. In Brazil, the Fiocruz/Oxford-AstraZeneca ChAdOx1-S COVID-19 vaccine is authorized for a 2-dose vaccination schedule with doses spaced 28 to 84 days apart. Sinovac Biotech’s CoronaVac vaccine has also received emergency use authorization (EUA) for a 2-dose regimen, with doses administered 28 days apart. Several other vaccines, based on different technologies, are either approved or anticipated to gain approval for combating SARS-CoV-2. Most of these vaccines are expected to be administered as a 2-dose series with matching doses. SCB-2019 contains a modified SARS-CoV-2 S-protein combined with an adjuvant. This protein is created by fusing the SARS-CoV-2 S protein into a trimeric structure using Trimer-Tag. SCB-2019 preserves this natural trimeric configuration and stimulates the production of neutralizing antibodies against SARS-CoV-2. The Trimer-Tag, which originates from a human protein, facilitates self-trimerization and allows for the fusion of this protein with others. When expressed in mammalian cells, these fusion proteins are secreted as linked homotrimers connected by disulfide bonds. Clemens et al. recently examined multiple formulations with SCB-209 vaccine, and they found that administering SCB-2019 as a booster to individuals previously primed with ChAdOx1-S resulted in elevated antibody levels against both the original SARS-CoV-2 strain and its variants, surpassing the immune response generated by a ChAdOx1-S booster of the same kind. The most substantial antibody responses were observed when using the 30-μg SCB-2019 + CpG + aluminum hydroxide formulation [129].

Human AdV 5-based vaccine delivering SARS-CoV-2 spike glycoprotein showed promising results in a Phase I trial with 108 participants, with rapid immune responses and no serious side effects (NCT04313127) [130]. Furthermore, a ChAdOx1-based vaccine is undergoing a Phase I/II trial, and it has been shown that most participants exhibit immune responses, including the production of neutralizing antibodies, following a single dose. Moreover, after receiving a booster dose, all participants demonstrated a 100% response. The effectiveness of HAdV 26.COV2.S vaccine by Johnson and Johnson was proven to be good and reduced the risk of hospitalization by 72% among immunocompetent adults without waning over 6 months postvaccination [131]. Recent clinical trial Phase IV study (NCT05409261) is evaluating immunogenicity and safety of COVID-19 vaccines, namely HAdV 26.COV2.S and NVX-CoV2373 post vaccination, focusing on assessing humoral immune response induced by vaccines in Mali.

Shingles (Herpes zoster): Shingles is a painful rash caused by the varicella-zoster virus that can occur anywhere on the body, which is the same virus that causes chickenpox. Once you have had chickenpox, the virus remains latent in body for life and can later resurface as shingles. While shingles is not life-threatening, it can be extremely painful. Getting vaccinated can reduce the risk of developing shingles [132]. In a preclinical study, Ulaszeska et al. developed a novel herpes-zoster vaccine utilizing the ChAdOx1 vector and evaluated the cellular and humoral immunogenicity of ChAdOx1-expressing VZV glycoprotein E (gE) in mice, compared with the licensed live HZ vaccine and recombinant HZ vaccine in a variety of dosing regimens. ChAdOx1-VZVgE enhanced T cell and antibody responses post-live HZ vaccine priming, especially with polyfunctional CD4+ and CD8^+^ T cells expressing multiple cytokines. This effect was consistent across both young and aged BALB/c mice. These results endorse the clinical advancement of ChAdOx1-VZVgE for preventing HZ in adults aged 50 and older, even if they have already received traditional vaccines [133]. An ongoing clinical trial (NCT05991427) involves 65 participants who will be given ChAdOx1-VZV, a chimpanzee AdV with adjuvanted zoster, intramuscularly. This Phase I trial is to evaluate the safety and immunogenicity of the recombinant AdV-based zoster vaccine.

Zika virus disease (ZVD): ZVD is caused by Zika virus, which is spread mostly via the bite of an infected Aedes species mosquito. Zika can be passed from a pregnant woman to her fetus. Infection during pregnancy can cause certain birth defects. Since the Zika virus outbreak in Brazil in 2015 [134], there has been significant ongoing research into the development of Zika virus vaccines. These vaccines incorporate genes encoding the membrane and envelope proteins of the Zika virus into the AdV vector genome [135,136]. The vaccine AdV26.ZIKV.M-Env, encoding Zika virus antigens, has been tested in mice and non-human primates. It induced strong and long-lasting immune responses, both humoral (antibodies) and cellular (T cells), against Zika virus in preclinical models. A single low-dose immunization protected mice from Zika virus, and non-human primates showed similar immune responses and complete protection against viremia after Zika virus challenge. In a clinical trial, an AdV 26-based vector vaccine successfully induced substantial levels of neutralizing antibodies against Zika virus without causing severe side effects [137,138]. Bacon et al. developed a thermally stable oral Zika vaccine, using a modified HAdV 5 to express Zika virus genes for the envelope and NS1 protein. It is formulated as an enteric coated capsule with OraPro. OraPro is a combination of sugars and modified amino acids that can overcome elevated temperatures and protects the integrity of AdV in extreme acidic conditions in the stomach. The vaccine was effective in inducing immune responses and reducing viral counts in mice and preventing viremia in non-human primates upon Zika virus challenge. This oral vaccine offers advantages over cold-storage vaccines that require injections [139]. This suggests AdV can be a good vector for a Zika vaccine if warranted.

B Meningococcal disease (MenB): Meningococcal group B disease is an uncommon but serious disease that is caused by a bacterial infection of the lining of the brain and spinal cord. It can also cause a severe infection of the blood called meningococcal septicemia [140]. Dold et al. recently discovered that their vaccine candidates generated strong immune responses with just one dose in various mouse models. They improved the vaccine and integrated it into the ChAdOx1 platform. This promising candidate is currently under development and could provide a one-shot solution for meningococcal disease [140].

Mycobacterium tuberculosis: Tuberculosis (TB) is one of the top 10 global causes of mortality and the primary cause of death due to bacterial infections, resulting in 1.5 million fatalities in 2020 alone. The World Health Organization (WHO) reports that approximately 25% of the world’s population carries Mycobacterium tuberculosis (M.Tb), the bacterium responsible for TB [141]. Researchers have developed ChAdOx1.85A, a chimpanzee AdV vaccine expressing the M.Tb antigen 85A, to minimize the impact of pre-existing neutralizing antibodies in humans. In mice primed with BCG, intramuscular immunization with ChAdOx1.85A showed high levels of cellular immune response and protective efficacy against M.Tb infection when combined with vaccinia Ankara virus expressing antigen 85A (MVA85A). A Phase I clinical trial (EMaBS) by the University of Oxford in 2020 confirmed ChAdOx1.85A vector’s safety and immunogenicity in healthy UK adults, with a Phase II trial that has recently finished (NCT03681860) with good outcomes. While trying to find increasing vaccine efficiency, researchers found out that AdV-based vaccines for TB may be most effective when given as booster shots. This has been demonstrated in various preclinical studies. AERAS-402, an HAdV35-based recombinant vaccine developed by Crucell and Aeras, demonstrated strong T cell immune responses and protection against tuberculosis in mice and rhesus macaques [142,143]. It was also found to be safe and immunogenic in Phase I clinical trials in healthy adults [144]. Subsequent trials expanded its target population to include various groups, including infants, adults with active or previous TB, latently infected individuals, and HIV-infected patients. Phase II trials targeting different populations have been completed (NCT02414828, NCT01017536, and NCT01198366), confirming the vaccine’s safety.

Different administration routes and combinations with other viral vaccines have also been tried. In a murine model, intranasal immunization with a recombinant ChAdOx1 vaccine expressing Rv1039c (PPE15) provided better protection than ChAdOx1.85A, warranting further clinical evaluation [145]. Another promising vaccine, AdCh68Ag85A, a chimpanzee adenovirus-68-vectored vaccine expressing Ag85A, induced strong T cell responses and protection against M.Tb infection in mice previously exposed to human adenovirus [146]. Additionally, it showed potential as a therapeutic vaccine, accelerating M.Tb clearance when administered via respiratory mucosal application alongside antibiotics in mice [141,147]. Validated clinical trials for combination vaccines also include AERAS-402 with ChAdOx1.85A [148] and FP85A (a recombinant avian poxvirus-vectored vaccine) [149]. Further vaccine research and development are warranted to successfully mimic this in humans for advanced clinical trial phases.

Malaria: Malaria is a serious and sometimes fatal parasitic infection caused by the Anopheles mosquito which feeds on humans. People who catch malaria are typically very sick with high fevers, shaking chills, and flu-like illness [150]. The deadliest malaria-causing parasite, *P. falciparum*, is the most common in Africa, while *P. vivax* is more common elsewhere in the world. Several preclinical studies have been conducted to develop vaccines against malaria, mostly based on rAdV 35 expressing CS protein of *P. yoelli* or *P. falciparum* sporozoites [151,152,153]. United States Army medical research and development command also tested AdV 5-based vaccines in clinical trials (NCT00392015 and NCT00870987). During these trials, the US Army attempted two approaches. In the first approach, they used a DNA-AdV vaccine that contained two antigens’ circumsporozoite protein (CSP), which is specific to the liver stage and apical membrane antigen-1 (AMA1), which is present in both the liver and blood stages. The goal was to prevent infection by targeting and eliminating developing parasites in the liver stage and provide protection against severe illness and potential fatality if blood stage infections occurred. In the second approach, they tested the NMRC-M3V-Ad-PfCA vaccine candidate, which utilized two adenovectors encoding CSP and AMA1, both from the 3D7 strain of P. falciparum in dose-escalation response. In both cases, the vaccines were found to be safe, immunogenic, and well tolerated but did not provide adequate protection against infection [154,155,156,157,158]. Currently 2 vaccines, namely RTS-S and R21, are recommended by the WHO. The R21 vaccine was recently recommended on October, 2023 for malaria prevention [159]. Coming back to AdV-based vaccines for malaria, a Phase II trial is currently ongoing, using chimpanzee AdV-based ChAdV63/MVA ME-TRAP, which encodes multiple epitope strings fused to the thrombospondin-related adhesion proteins (NCT03947190). The trial will test the safety and efficacy of chAdV-63 based malarial vaccine with R21/Matrix-M adjuvant in Kenyan adults.

Leishmaniasis infection: Leishmaniasis is caused by protozoan parasites which are transmitted by the bite of infected female phlebotomine sandflies. It is widespread across 95 countries, leading to substantial morbidity and mortality. The World Health Organization recognizes the leishmaniases as some of the most significant global neglected diseases associated with poverty, with over one billion people at risk of infection, with one million new cases and over 20,000 deaths reported each year [160,161]. While there is substantial experimental and epidemiological evidence suggesting that vaccination could prevent leishmaniasis, there is currently no approved vaccine for human use. Researchers have developed ChAdV 63-KH, a third-generation adenovirus-vectored vaccine based on the well-established ChAdV 63 platform [162]. ChAdV-vectored vaccines are known for their strong T cell and antibody responses in humans and are suitable for large-scale production under GMP standards. ChAdV 63-KH contains two Leishmania antigens, KMP-11 and HASPB, both demonstrating vaccine efficacy in animal models. KMP-11 is a conserved protein with CD8^+^ T cell epitopes, while HASPB exhibits polymorphic repeats [163,164,165]. To enhance cross-isolate coverage, a synthetic KH fusion gene was added for ChAdV 63-KH, making it a potential pan-leishmaniasis vaccine. A UK first-in-human trial (ISRCTN: 07766359) demonstrated that a single dose of ChAdV 63-KH vaccine was safe and well tolerated and stimulated strong innate and cell-mediated immune responses [166]. The LEISH2a trial, a Phase IIa study (NCT02894008), was conducted in Sudanese patients. Patients received a single intramuscular ChAdV 63-KH vaccine injection, with a primary focus on safety and secondary assessments of clinical response and immunogenicity. The vaccine demonstrated minimal adverse reactions and elicited strong innate and cell-mediated immune responses, as shown via transcriptomics and ELISpot assays. Blood transcriptomic analysis identified immune markers predictive of more than 90% clinical improvement [161]. A more recently completed Phase IIb trial (NCT03969134) was conducted to assess the therapeutic efficacy and safety of ChAdV 63-KH in patients with persistent PKDL in Sudan. ChAdV 63-KH vaccine was intramuscularly administered with a dose of 7.5 × 10^10^ VPs [167]. The therapeutic benefits of vaccination with ChAdV 63-KH in PKDL patients are expected in 2023. If the therapeutic effectiveness of ChAdV 63-KH in PKDL is confirmed, it should be contemplated for further assessment in different manifestations of leishmaniasis [167].

Lynch Syndrome: Lynch syndrome is an inherited cancer syndrome linked to a genetic inclination toward various cancer forms. Individuals with Lynch syndrome face an elevated risk of specific cancer types. It is also recognized as hereditary non-polyposis colorectal cancer (HNPCC) [168]. An ongoing Phase IIb trial (NCT05419011) examines whether a multitargeted HAdV 5-based (Tri-Ad5) vaccine, in conjunction with N-803, can prevent colon and other cancers in individuals with Lynch syndrome. Tri-Ad5 contains substances found in precancerous and cancer cells, potentially stimulating the immune system to recognize and eliminate future cells with these proteins. An IL-15 super agonist, N-803, enhances immune responses to vaccines, potentially reducing the risk of colon and other cancers in Lynch syndrome participants when combined with Tri-Ad5. Another Phase Ib/II trial (NCT05078866) is assessing Nous-209 vaccine safety. Nous-209 contains man-made copies of neoantigens produced by cells with genetic errors. The doses will first be an adenovirus tumor-specific neoantigen priming vaccine GAdV-209-FSP (GAdV 20-209-FSPs) (1 prime), and the second dose will be the MVA tumor-specific neoantigen boosting vaccine MVA-209-FSP (MVA-209-FSPs). This trial may help researchers determine whether receiving Nous-209 vector has an effect on the development of polyps or tumors in the colon.

Cocaine dependence: Cocaine is a highly addictive tropane alkaloid that acts as a central nervous system stimulant. As an extract, it is mainly used recreationally, and often illegally, for its euphoric and rewarding effects. Cocaine abuse is a global health crisis. Acute cocaine toxicity necessitates immediate treatment to address issues such as rapid heartbeat, irregular heart rhythm, high blood pressure, and coronary artery spasms in order to prevent serious outcomes such as heart attacks, strokes, and fatalities [169,170]. Currently 150 participants are enrolled in a Phase I clinical trial (NCT02455479) for a safety study of a disrupted AdV (dAd5GNE) cocaine vaccine. The vaccine prevents cocaine from reaching the brain by using a molecule called GNE, which is linked to a disrupted HAdV 5 capsid proteins. It stimulates the immune system to produce anti-cocaine antibodies that bind to cocaine molecules, preventing them from entering the brain. This approach has been effective in preclinical studies in mice, rats, and nonhuman primates, resulting in high levels of anti-cocaine antibodies [171].

## 7. Addressing Key Challenges for Enhanced Adenovirus Vector Performance

### 7.1. Pre-Existing Humoral and Cellular Immunity

Despite the availability of various rAdV vectors for preclinical uses, research has revealed significant challenges due to widespread pre-existing immunity against common HAdV serotypes. HAdV neutralizing antibodies (nAbs) significantly hinder viral vectors from effectively targeting cells and reducing adaptive immune responses [172]. This immune response remains a major hurdle in rAdV gene therapy, even though rAdV vector-based vaccines and oncolytic therapies can benefit from it. Consequently, controlling rAdV vector-induced innate immune responses is crucial for the success of these approaches. Finding an adenovirus serotype that is rarely encountered by humans could be considered as a potential choice for individuals already infected with adenovirus. To counter pre-existing immunity against AdV vectors, various strategies are being employed. Modifying the adenovirus capsid to either evade or reduce the impact of nAbs or to facilitate the virus’s tropism toward specific cell types is a highly intriguing field currently under active investigation in both academic and pharmaceutical circles. Efforts to bypass the existing immune response and change the virus’s preferred targets have been explored with hybrid capsid proteins [57,173].

First, less-common human serotypes, such as HAdV 2, HAdV 26, and HAdV 35, which have lower seroprevalence, are being used as vectors to reduce the impact of pre-existing immunity. However, these vectors have been shown to be less effective in inducing an immune response compared to the commonly used HAdV 5 [174]. Additionally, non-human AdV vectors derived from animal sources, such as bovine (BAdV), canine (CAdV), chimpanzee (ChAdV), ovine (OAdV), porcine (PAdV), and fowl (FAdV), have been developed to minimize cross-reactive immunity. Among these, chimpanzee-derived AdV vectors are widely used due to lower levels of neutralizing antibodies in human circulation [175]. So far, more than 10 clinical trials using ChAdV 3-derived vaccines have proven the safety of these replication-deficient vectors [176]. Furthermore, high-capacity TGAdV vectors can achieve long-term transgene expression in the body by reducing host immune responses against residual viral proteins [49,177,178]. Currently a clinical trial (NCT05315856) is accessing humoral antibody and cytokine kinetics after vaccination with either BNT162b2 (mRNA-based) or ChAdOX1 nCoV-19 (chimpanzee AdV-based) vaccine and other factors influencing the vaccine immunogenicity. Recently D’Alise et al. generated Ad-9D9, an AdV vector encoding a murine anti-CTLA-4 monoclonal antibody (Ad-9D9) as a genetic adjuvant for adenovirus-based vaccines. They demonstrated that Ad-α-CTLA-4′s adjuvant effect is not tied to the vaccine’s antigen itself. Instead, it enhanced the immune response and effectiveness of an adenovirus-based polyepitope vaccine containing tumor neoantigens. The study shows that using AdV encoded adjuvant (AdEnA) alongside an AdV-encoded antigen vaccine boosts immune responses to both viral and tumor antigens, offering a promising strategy for creating more effective genetic vaccines [179].

Another way to bypass pre-existing immunity is to try different routes of administration for different doses [2]. Depending on how they are given, AdV vaccines, gene therapy, and oncolytic therapy vectors can encounter different components in the bloodstream such as blood cells, immune maintenance cells, and various other cell types. Local administration, such as (intramuscular or intratumoral), is generally safer than injecting into a vein as demonstrated by multiple trials. However, it is not always possible. Some amount of vector may still find its way into the bloodstream, especially in case of inflammation event. Current SARS-CoV-2 vaccines, especially mRNA-based ones, are effective in generating strong humoral and cellular immunity, preventing severe disease. However, they have limited efficacy in preventing asymptomatic to mild infections and transmission, especially when dealing with SARS-CoV-2 variants of concern (VOCs). Systemic mRNA vaccination alone in a mouse model led to weak respiratory mucosal neutralizing antibodies, especially against SARS-CoV-2 Omicron BA.1.1. Combining systemic mRNA vaccination with mucosal adenovirus-S immunization induced strong neutralizing antibodies against both the original virus and the Omicron BA.1.1. variant [180].

### 7.2. Manufacturing Bottlenecks

The demand for substantial quantities of clinical-grade rAdVs—ranging from 10^12^ to 10^13^ viral particles per patient, or 10^10^ to 10^11^ plaque-forming units per patient—underscores the necessity for highly effective and well-established large-scale production processes compliant with cGMP [181]. The optimization of the manufacturing process must exhibit exceptional scalability. Otherwise, as it transitions to larger scales, process failures may occur, resulting in extended timelines for further optimization and subsequently elevated costs. To establish dependable bioprocessing techniques for rAdV production, it is imperative to possess contemporary insights into the entire production systems and to comprehend the pivotal factors that influences rAdV yield and purity. Reducing product and process related impurities are the most important part of any industrial biomanufacturing operations including in AdV vector manufacturing.

Efficient production of rAdV vectors at higher titers relies on advanced cell-culture techniques. The cell environment in rAdV production has been thoroughly examined due to its intricate nature. It requires sustaining cell growth while achieving superior rAdV yield. This environment involves multiple factors such as healthy growth medium composition, physicochemical conditions such as pH, optimized feeding strategies, and cellular stresses induced via various cell-culture methods. While adherent cell cultures suffice for small-scale production, larger applications require adapted cell lines in serum-free suspension conditions. Adherent cells utilize microcarriers in bioreactors. Harvesting adherent infected cells involves trypsinization or detachment. While in suspension cell-bioreactors, infected cells are recovered via centrifugation or filtration. An integrated process is essential for the optimization of large-scale rAdV production using serum-free suspension-cultured HEK293 cells (20–500 L). Efforts focus on fine-tuning operating conditions such as bioreactor infection, feed-rate, and final harvest time-points. Recombinant AdV-infected cells are cultured until full lysis, and then cell debris with spent media containing supernatant are collected for further processing in downstream purification. Detailed time-course analysis optimizes perfect harvesting conditions and time-points.

**Figure 4:**
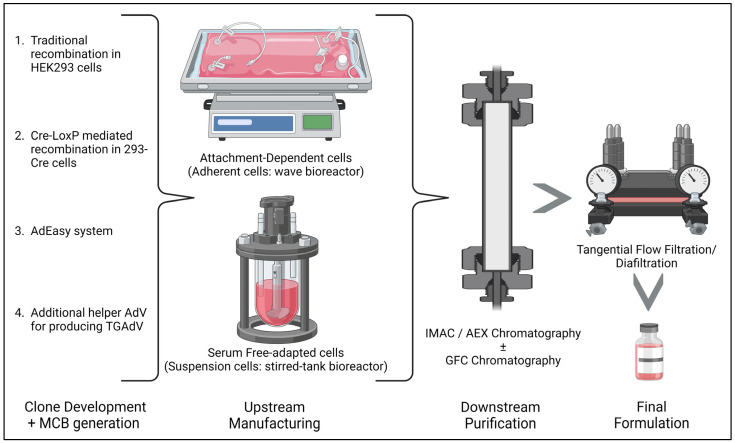
Represents simplified version of large-scale operation for manufacturing adenovirus vector using specified platform. During clone development, a master cell bank (MCB) and Master Virus Seed is generated, then a working cell bank can be made for further use. This initial stage changes a great deal depending on the process development required for the specific generation of AdV vector. As optimized in this initial stage, the upstream manufacturing platform is chosen to be performed by infecting in either adherent cell line format or in suspension cell line format. The purification and final polishing of the vector is conducted by using combination of optimized chromatography steps such as immobilized metal-ion affinity chromatography (IMAC), anion exchange chromatography (AEX), and/or gel filtration chromatography (GFC). Depending on the required final concentration of vector in specific formulation buffer for increased stability, tangential flow filtration and diafilteration (TFF) can be performed before the fill-finish process. Created with BioRender.com (accessed on 1 December 2023).

Adenovirus purification starts with cell lysis, often achieved via freeze/thaw cycles or pressure-based methods, releasing virus particles along with host cell and viral DNA and RNA into the spent media/crude lysate. Nucleases such as benzonase digest these nucleic acids, but removing residual Benzonase can be challenging. Some purification methods require detergents and protease treatment during the lysis step. Recombinant AdV vector purification at the lab scale has historically used centrifugation with a cesium chloride gradient, but its toxicity makes it unsuitable for clinical use. Modern protocols combine precipitation, aqueous two-phase extraction, and iodixanol gradient centrifugation, which improves safety by reducing toxic impurities and minimizing virus inactivation. However, these lab-scale methods are inefficient for large-scale production. For the efficient purification of recombinant AdV gene therapy-related vectors, affinity chromatography and anion exchange techniques are often employed [182,183,184]. In both methods, viral vector binding and eluting play a very important role in maintaining capsid integrity. These elution parameters are based on specific salt concentrations and pH levels of elution buffer. Multiple conditions must be explored and optimized very carefully, as there is a fine line between recovery and purity of the eluted sample. Removal of extracellular host cell DNA (HC-DNA) and host cell proteins (HCPs) with minimal endotoxin levels in drug substance (DS) is desired outcome, chromatography can be very useful to reduce HC-DNA and HCPs with optimized wash steps. Size-exclusion chromatography or tangential flow filtration (TFF) step need to be added for final finishing stage as a polishing step. However, prior to size-exclusion chromatography, viral particles must be concentrated via ultrafiltration, which can lead to significant viral loss due to particle aggregation near the membrane. This tandem ion-exchange/size-exclusion chromatographic process successfully achieves 99% pure adenovirus recovery from large-scale suspension production. Some of the AdV-based vaccines typically have a dose of 10^11^–10^12^ vp/mL for IM administration [181]. For oncolytic therapies, dose concentration varies depending on the route, target tissues and stage of treatment e.g., for treating a high dose therapy may need concentration of 10^14^ vp/mL considering minimal spill over towards surrounding tissues. This dose concentration of final drug substance should also be considered while process optimization and scale up plan as higher concentration leads to aggregation in improper formulations. Figure 4 illustrates a simplified large scale rAdV vector manufacturing strategy.

Recently, TGAdV has found diverse applications demanding sustained transgene expression, presenting clear advantages over first-generation AdV vectors. However, manufacturing TGAdV in clinically relevant quantities remain very challenging. Existing TGAdV systems face two key limitations: limited virus production and possibility of contamination with residual helper viruses. Traditionally, the latter issue can be mitigated via Cesium Chloride (CsCl) ultracentrifugation based on genome size differences, but this method is not at all suitable for large-scale clinical vector production. Applying modern chromatographic purification techniques developed for FGAdV is complex due to possible helper virus contamination in TGAdV vector purification. A future objective for the helper-dependent vector production system is to reduce the quantities of helper virus via packaging gene-complementing cell lines and targeted recombinase-mediated deletion of the packaging signal which would help to mitigate residual virus contamination. Decisions regarding mammalian cell specifications, cell-culture characteristics, cell density, multiple viral parameters including MOIs, and vector construction must align with the final viral product identity for optimal AdV production with required viral vector concentration. Enhanced comprehension of viral amplification and cell physiology will help create a more efficient viral cell system for high-specific productivity at high cell densities. As the field moves forward, the knowledge gained from developing FGAdV production will be valuable for enhancing third generation AdV production, which has superior *in vivo* potential for gene therapy applications.

### 7.3. Viral Vector Characterization Dilemma

Throughout the entire manufacturing process, including viral amplification, purification, and formulation, a thorough measurement is essential to assess and regulate process parameters. Quantification serves multiple purposes, such as overseeing viral amplification via the MOI (multiplicity of infection, the number of infectious viral particles per cell during infection) and monitoring production yields. To standardize the diverse quantification methods, the FDA, along with multiple partners, initiated the development of an HAdV 5 reference material (ARM) [185]. Initially, the aim was to create and comprehensively examine an HAdV stock using representative assays to aid in validating new methods. This encouraged sharing standard operating procedures (SOPs) among various development and manufacturing groups, leading to the adoption of best practices in the field. However, the FGAdV reference material did not become a universal standard for all HAdV generations, despite multiple attempts to adapt it. Since the development of FGAdV ARM, there have been multiple attempts to generate ARM for multiple HAdVs, including the latest TGAdV. Recently, Chen et al. studied a digital PCR method and development of reference material for HAdV 40 and 41 [186]. There is still confusion regarding standard ARM usage across multiple HAdV generations. More guidance and common understanding are required to establish a standard source of ARMs across multiple generations to normalize it across fields and species. To accurately quantify rAdV originating from multiple groups and animal origins, many studies have recently been completed using dPCR, ddPCR, or UHPLC-MS/MS [187,188,189,190,191]. We need an optimized and largely accepted rAdV quantification technique which would be easier to adapt from academia to industry to make it more versatile. This would result in more adapted and commonly understood techniques for both viral particle assays and infectious viral particle assays.

### 7.4. Seroepidemiological Data

Seroepidemiology is a valuable tool in public health and animal health research. It helps to determine the frequency of infections, evaluate efficient immunization efforts, and investigate other past outbreaks when a novel virus is found in specific area. When researchers collect blood samples from people several weeks apart, the appearance of antibodies or an increase in specific antibody levels in the second sample shows recent infection [192]. Numerous adenovirus-related seroepidemiological research and clinical trials rely on findings from over two to four decades ago. Seroepidemiological studies have always mainly focused on North America, western Europe, China, and Japan. However, there is a lack of comprehensive seroepidemiological data for South America, Australasia, and many African countries. For instance, in Africa and Asia, HAdV-D26 seroprevalence appears to be relatively high, while it is very low in North America and Europe. Additionally, HAdV-B35 seroprevalence is generally low across the world, based on some of limited available studies [4]. Antibodies against certain NHP AdVs can be found in humans, possibly due to cross-reactivity. The analytical methods and techniques (discussed above) used in seroepidemiological studies vary significantly among different research efforts, making comparisons challenging at times. Determining HAdV seroprevalence also poses challenges, primarily due to increased international travel and complex medical, political, and ever-changing social situations in certain areas around the world. There are some recent studies conducted around the world for making the data available to the scientific community as whole [193,194,195,196,197,198,199]. More research efforts need to be undertaken for global seroepidemiological studies, the advancement of basic research on vector biology and investigation into AdV interaction with the host’s existing immunity [4].

## 8. Recent Progress in the Field

Virus-based therapies, once hampered by limited available knowledge of viral biology, have witnessed a transformative evolution over the past four decades. This burgeoning knowledge has given rise to a diverse array of promising viral vector-based strategies for combatting genetic diseases and providing multiple treatment options to human population. These viral vectors comprise a few essential components, which could be altered and optimized: the protein capsid or envelope which dictates tissue and cell targeting specificity (serotype specificity), the therapeutic transgene, and the regulatory cassette/promoter governing transgene expression in specific tissues. Each viral vector platform demands unique design considerations, presenting distinctive strengths and limitations that offer numerous effective treatment avenues for human diseases. However, in the case of adenovirus, generating three generations of AdVs may prove insufficient due to over half of the human population being seropositive. However, placing exclusive reliance in genetic modifications on AdV vector capsid assembly may not yield significant progress without further research efforts. The following approaches might be helpful in making AdV vectors more efficient. Modifying RGD-directed structural AdV might be an alternative combinatorial peptide display which would lead to higher specificity and efficient endocytosis, but it might have to assess the safety profile of this approach related to the immune response as well, like discussed previously for rAAV related optimizations [200]. A more promising approach involves integrating the promoter regions of genes highly active in cancer cells, such as human telomerase reverse transcriptase and cyclooxygenase 2, into locations that govern adenovirus replication gene expression [201,202]. This strategic adaptation empowers oncolytic viruses to replicate exclusively within cancerous cells, offering a potent avenue in cancer therapy. Recently, Bates et al. showcased precise glioblastoma-specific transgene expression using bio-engineered rAdV constructs. This suggests that the incorporation of pseudotyping and tumor-specific promoter strategies might facilitate the creation of more effective treatments tailored to glioblastoma, and potentially for other cancers as well [203].

Dickson et al. studied the role of vaccination with an AdV vectored vaccine in a SARS-CoV-2/K18-hACE2 mouse infection model. Mice were given the AdCOVID vaccine either intramuscularly (IM) or intranasally (NAS) and then exposed to SARS-CoV-2; it was demonstrated that those vaccinated NAS had stronger cellular and mucosal antibody responses. Furthermore, NAS vaccination led to better control of the virus in the blood and protection against lethal infection when compared to IM vaccination. In a unique transmission test, NAS vaccination also reduced viral spread to unvaccinated mice living together compared to the IM route [204]. This can lead to alternate routes of administration for AdV-derived vaccines in future, depending on disease type. A first-in-human study of a COVID-19 vaccine will be investigating the effects of the SC-Ad6-1 vaccine given the the intramuscular and intranasal routes or inhalation in healthy volunteers (NCT04839042). Many more clinical studies are required in this aspect. In terms of manufacturing and purifying adenovirus vectors, no single production system is suitable for every generation of AdV. There are many scalability optimizations and comparative analysis already conducted in the AAV manufacturing field; a similar approach is required with AdV vectors to increase yield and purity [205,206]. As we move towards the future, we need to learn from past and recent approvals of three AdV-based vector vaccines, namely AdV 26-ZEBROV against Ebola, AdV 26-COV2-S and ChAdOx1-S against COVID-19 gives big boost to the AdV-based vaccine field [207,208,209]. The only approved adenovirus vector for gene therapy is Nadofaragene firadenovec, known as Adstiladrin or rAdV-IFNa/Syn3. It is a non-replicating adenovirus vector derived from AdV 5, with the inclusion of the human IFN-α2b gene [210,211,212,213]. It received approval in the United States in December 2022 for treating non-muscle-invasive bladder cancer (NMIBC). The first patient was dosed on September, 2023 while writing this article [214]. Moments such as this invigorate and motivates researchers to conduct more research work for the betterment of human beings.

## 9. Conclusions

Over the past few decades, there has been a fluctuating trend in the number of clinical trials involving adenovirus vectors. Since the last pandemic, adenoviruses are emerging onto the clinical landscape as suitable vectors for gene therapy, for cancer therapy, and especially for vaccines. The ongoing success of adenoviruses as vaccine carriers is not only because they can trigger strong and lasting immune responses in both the blood and cells, but also because they can provoke these responses at the points of viral entry. The surge in daily increments of trials exploring various recombinant adenoviral vectors reflects a continuous and growing interest in this research domain. These ongoing clinical efforts are exploring newer types of vectors which aim to expand the therapeutic possibilities of gene therapy in addressing adenovirus-related issues as well. However, advancing adenovirus-based gene therapies faces biological, manufacturing, and regulatory challenges which demands significant contributions from preclinical, clinical, and commercial sectors. Nonetheless, scientific progress in viral vector engineering, disease related genomics understanding, and fundamental gene editing using CRISPR-Cas system is propelling viral gene therapies into a new era. More clinical and preclinical research still needs to be conducted for making adenovirus vectors more common and trustworthy as AAV vectors. The field of viral vector-based gene therapy is expected to remain highly active in clinical research, with the anticipation of more adenovirus vector-based products entering the market.

## Figures and Tables

**Table 1:** Functions of key genes in the adenovirus genome.

Adenovirus Related Genes	Functions
Capsid proteins II, III, IIIa, IV, VI, VIII, and IX	Structural proteins for capsid
Core proteins V, VII, X	Structural proteins for capsid
CR1β	Membrane glycoprotein, helps modulate the host immune response
E1A	Most important early gene, activates transcription of a number of viral genes as well as genes of the host cells
E1B55K	Binds to and inactivates p53, to block p53-mediated functions in the cell
E2A and E2B	Participates in replication of viral DNA
E3 RIDα and E3 RIDβ	Membrane proteins, plays role to prevent apoptosis
E3 gp19K	Membrane glycoprotein, blocks class I MHC protein insertion in the host cell membrane to prevent T cell recognition of viral infection
E3 14.7K	Defends the virus from host antiviral responses
E4 transcription unit	Multifunctional viral proteins regulate viral and host-cell gene expressions
IVa2, 52K, L1, and 100K	Encapsidation proteins, helps in proper assembly of viral capsids
L3 protease	Cleave precursor polypeptides of pTP, pVI, pVII, pVIII, and IIIa to produce the mature viral proteins
Terminal protein TP	Covalently attached to 5′ ends of the DNA, plays critical role in replication and long-term infectivity

**Table 2:** Selective ongoing clinical trials using recombinant adenoviruses as vector.

SN	NCT Identifier	Trial Stage	Conditions	Transgene/Strategy	Route	Enrol.
1	NCT05124002	Phase IV	Intrahepatic Cholangiocarcinoma	rHAdV 5 (H101) + HAIC	ITU	66
2	NCT04452591	Phase III	Non-Muscle Invasive Bladder Cancer (NMIBC)	DDM in engineered oncolytic rAdV (CG0070) + n-dodecyl-B-D-maltoside	IVE	110
3	NCT03780049	Phase III	Hepatocellular carcinoma	Hepatic artery infusion chemotherapy ± H101	IV	304
4	NCT02928094	Phase III	Refractory Angina Due to Myocardial Ischemia	HAdV 5 + FGF-4	ICOI	160
5	NCT05664139	Phase II	Liver Metastases from Malignant Melanoma	HAdV 5 + PD-1 mAb + Nab-paclitaxel	ITU/IV	30
6	NCT04111172	Phase II	Gastrointestinal Adenocarcinoma	HAdV 5 + F35-hGCC-PADRE	IM	81
7	NCT03947190	Phase II	Malaria Vaccine	ChAdV 63/MVA ME-TRAP + R21/Matrix-M	ID/DVI	64
8	NCT05419011	Phase II	Lynch Syndrome	CEA/MUC1/Brachyury (TRI-AdV 5)+ IL-15 Superagonist N-803	ITU	186
9	NCT05872841	Phase II	Primary Hepatocellular Carcinoma	H101 Combined + TACE	ITU	38
10	NCT05564897	Phase II	Bladder Cancer	Oncolytic AdV + PD-1 inhibitor (Camrelizumab)	IVE	25
11	NCT05419011	Phase II	Lynch Syndrome	Tri-AdV 5 + IL-15 superagonist nogapendekin alfa inbakicept (N-803)	SC	186
12	NCT05234905	Phase II	Cervical Cancer	H101 + Camrelizumab	ITU	55
13	NCT01913106	Phase II	Prostate Cancer	AdV/RSC-TK + Brachytherapy	ITU	25
14	NCT03039751	Phase II	Refractory Angina Pectoris	AdV + VEGF-D	IMCD	180
15	NCT04095689	Phase II	Triple Negative Breast Cancer	Docetaxel + Pembrolizumab + AdV/IL-12	ITU	30
16	NCT04495153	Phase II	Non-Small Cell Lung Cancer	Aglatimagene besadenovec (CAN-2409 + prodrug)	ITU	86
17	NCT04416516	Phase II	Basal Cell Carcinomas/Basal Cell Nevus Syndrome	ASN-002 + Hh inhibitor vismodegib	ITU	18
18	NCT04739046	Phase II	Pancreatic cancer	HAdV 5-yCD/mutTKSR39rep-ADP (Theragene)	ITU	12
19	NCT05441410	Phase I/II	Malaria	ME-TRAP + ChAdV 63	IM	30
20	NCT04097002	Phase I/IIa	Prostate Cancer	Improved AdV 5 (ORCA-010)	ITU	24
21	NCT05078866	Phase Ib/II	Lynch Syndrome	AdV neoantigen priming vaccine GAdV-209-FSP + MVA tumor-specific neoantigen-boosting vaccine MVA-209-FSP	IM	45
22	NCT04673942	Phase I/II	Refractory Solid Tumors	Replicative AdV 5 + TGF-β receptor-immunoglobulin Fc fusion trap (AdAPT-001)	ITU	79
23	NCT03754933	Phase I/II	Head/Neck Cancer	rAdV expressing *E. coli* Purine nucleoside phosphorylase + fludarabine phosphate (Ad/PNP)	ITU	10
24	NCT02749331	Phase I/II	Neuroendocrine Tumors	rAdV AdVince (CgA-E1AmiR122)	HAI	35
25	NCT02705196	Phase I/II	Pancreatic Cancer	rAdV + TMZ-CD40L + 4-1BBL (LOAd703)	ITU	55
26	NCT05617040	Phase I/II	Prostate Cancer	ChAdOx1-PCAQ + MVA-PCAQ + PSA + PAP + STEAP1+ 5T4	IM/IV	137
27	NCT05914935	Phase I	Malignant Tumors; Glioblastoma	AdV + rL-IFN	ITU	6
28	NCT04695327	Phase I	Solid Tumors	TNFα + IL-2 + rAdV (TILT-123)	ITU	18
29	NCT05180851	Phase I	Head and Neck Cancer; Melanoma; Ovarian/Cervical Carcinoma; Lung Cancer	rAdV L-IFN (YSCH-01)	ITU	19-28
30	NCT04053283	Phase I	Metastatic or Advanced Epithelial Tumors	Tumor-selective transgene expressing AdV (NG-641)	IV	186
31	NCT05271318	Phase I	Ovarian Cancer	rAdV TILT-123 + pembrolizumab [AdV 5/3-E2F-d24-hTNFa-IRES-hIL2]	ITU	29
32	NCT05222932	Phase I	Melanoma; Head and Neck Squamous Cell Carcinoma	rAdV (TILT-123) + avelumab + anti-PD1 (L) (AVENTIL) [AdV 5/3-E2F-d24-hTNFa-IRES-hIL2]	ITU	15
33	NCT05165433	Phase I	Metastatic or Advanced Epithelial Tumors	NG-350A vector + pembrolizumab (tumor-selective anti-CD40)	IV	198
34	NCT05686798	Phase I	Progressive Astrocytoma; GBM; Brain Tumor	AdV 5-yCD/ mutTKSR39rep-ADP	ITU	18
35	NCT05043714	Phase I	Metastatic or Advanced Epithelial Tumors (NEBULA)	NG-641 vector + nivolumab (NG-641)	IV	30
36	NCT04217473	Phase I	Advanced Melanoma	TNFalpha + IL 2 coding rAdV TILT-123 (TUNINTIL)	ITU	15
37	NCT03896568	Phase I	High-Grade Glioma	MSC-DNX-2401 (AdV 5-DNX-2401)	IA	36
38	NCT03740256	Phase I	Advanced HER2 Positive Solid Tumors	HER2-specific CAR-T + ChAdVEC	ITU	45
39	NCT03896568	Phase I	Recurrent High-Grade Glioma	BM-hMSCs + DNX-2401 (MSC-DNX-2401)	IA	36
40	NCT04053283	Phase I	Metastatic Cancer, Epithelial Tumor	rAdV + FAP-TAc antibody/ CXCL9/CXCL10/IFN-α (NG-641)	IV	186
41	NCT03284268	Phase I	Refractory Retinoblastoma (RTB)	rAdV (VCN-01)	IVE	13
42	NCT02455479	Phase I	Cocaine-Dependent Individuals	Disruptive dAd5GNE	IV	15
43	NCT05076760	Phase I	Non-Small Cell Lung Cancer	rAdV (MEM-288) + IFNβ + rCD40-ligand	ITU	18
44	NCT03878121	Phase I	HIV	ADV 4-HIV envelope vaccine vectors [AdV 4-Env145NFL + AdV 4-Env150KN + VRC-HIVRGP096-00-VP (Trimer 4571)]	IN/IM	300
45	NCT04839042	Phase I	COVID-19 Vaccine	AdV 6 vector (SC-AdV 6-1)	IM/IN/ IH	190
46	NCT05526183	Phase I	COVID-19 Vaccine	AdV 5 CoVacHGMix (with equal amounts of CoVacHGA1320, CoVacHGB420, CoVacHGC720 and CoVacHGD1480	IM	36
47	NCT05717699	Phase I	Intrinsic Pontine Glioma	AdV 5-TD-nsIL12 + human non-secretory interleukin-12	ITU	18
48	NCT05717712	Phase I	Intrinsic Pontine Glioma	AdV 5-TD-nsIL12 + human non-secretory interleukin-12	ITU	18
49	NCT03546361	Phase I	Non-Small Cell Lung Cancer	CCL21-Gene modified dendritic cell (rAdV -CCL21-DC) + pembrolizumab	ITU + IV	24
50	NCT05991427	Phase I	Zoster Disease	ChAdOx1-VZV	IM	65

**Table 3:** Adenovirus-based clinically approved vectors.

SN	Name	Sponsor	AdV Type	Payload/ Antigen	Application
1	Jcovden	Johnson & Johnson (Solothurn, Switzerland)	AdV 26	CoV2-S spike protein	COVID-19 vaccine
2	Convidecia	CanSino Biologics Inc. (Tianjin, China)	AdV 5	SARS-CoV-2 spike protein	COVID-19 vaccine
3	Vaxzevria / Covishield	AstraZeneca/ University of Oxford (Oxford, UK)	ChAdV	ChAdOx1-S spike protein antigen	COVID-19 vaccine
4	Sputnik V	Gamaleya Research Institute (Moscow, Russia)	AdV 26 (prime) + AdV 5 (boost)	Spike protein (S) antigen	COVID-19 vaccine
5	Zabdeno	Johnson and Johnson (Solothurn, Switzerland)	AdV 26	Ad26.ZEBOV	Ebola Vaccine
6	Adstiladrin	Ferring Pharmaceuticals (Parsippany-Troy Hills, NJ, USA)	AdV 5	Nadofaragene firadenovec-vncg with IFNα2b	Gene Therapy for Bladder Cancer
7	H101/Oncorine	Shanghai Sunway Biotech (Shanghai, China)	AdV 5	Tumor-specific	Cancer Therapy for Nasopharyngeal cancer
8	Gendicine	Shenzhen SiBiono GeneTech (Shenzhen, China)	AdV 5	Anti-p53	Cancer Therapy for Head and neck cancer
